# Real-world longitudinal data collected from the SleepHealth mobile app study

**DOI:** 10.1038/s41597-020-00753-2

**Published:** 2020-11-27

**Authors:** Sean Deering, Abhishek Pratap, Christine Suver, A. Joseph Borelli, Adam Amdur, Will Headapohl, Carl J. Stepnowsky

**Affiliations:** 1 Health Services Research & Development, VA San Diego Healthcare System, San Diego, CA 92161 USA; 2 Sage Bionetworks, Seattle, WA 98109 USA; 3 American Sleep Apnea Association, Washington, DC 20001 USA; 4 Department of Medicine, University of California at San Diego, La Jolla, CA 92037 USA

**Keywords:** Quality of life, Outcomes research

## Abstract

Conducting biomedical research using smartphones is a novel approach to studying health and disease that is only beginning to be meaningfully explored. Gathering large-scale, real-world data to track disease manifestation and long-term trajectory in this manner is quite practical and largely untapped. Researchers can assess large study cohorts using surveys and sensor-based activities that can be interspersed with participants’ daily routines. In addition, this approach offers a medium for researchers to collect contextual and environmental data via device-based sensors, data aggregator frameworks, and connected wearable devices. The main aim of the SleepHealth Mobile App Study (SHMAS) was to gain a better understanding of the relationship between sleep habits and daytime functioning utilizing a novel digital health approach. Secondary goals included assessing the feasibility of a fully-remote approach to obtaining clinical characteristics of participants, evaluating data validity, and examining user retention patterns and data-sharing preferences. Here, we provide a description of data collected from 7,250 participants living in the United States who chose to share their data broadly with the study team and qualified researchers worldwide.

## Abstract (summary)

**Table:** 

Measurement(s)	Demographic Data • Alcohol Consumption • Smoking Behavior • Socioeconomic Factors • Personal Medical History • Sleepiness • Sleep Quality • Objective Alertness
Technology Type(s)	Survey • Epworth Sleepiness Scale Questionnaire • Pittsburgh Sleep Quality Index • Psychomotor Vigilance Task 3-Minute Version (PVT-B)
Sample Characteristic - Organism	Homo sapiens
Sample Characteristic - Location	United States of America

Machine-accessible metadata file describing the reported data: 10.6084/m9.figshare.12647108

## Background & Summary

The Center for Disease Control (CDC) has deemed insufficient sleep to be a “public health epidemic” based on evidence that over one-third of American adults are not getting enough regular sleep^1,2^. More than 40 million Americans suffer from over 80 different sleep disorders, and another 20–30 million suffer from intermittent sleep problems^3^. Sleep accounts for one-quarter to one-third of our day and has a significant effect on health, well-being, and daytime performance^4^. Poor sleep comprising both short and long sleep periods is associated with impaired cognitive performance^5^. Short sleep is linked with seven of the top fifteen causes of death in the United States^6^, decreased workplace productivity^7^, and an estimated $680 billion in economic losses across five major countries^8^.

The diagnosis and treatment of sleep disorders has been an integral part of medical practice for the last several decades, but a more holistic approach focused on health and wellness has recently started to emerge. This integrated approach is known as “sleep health”, and it focuses on studying sleep in the general population^9^. Sleep health research is beginning to demonstrate that many facets of sleep are related to important health outcomes^10^.

Traditional sleep research methods utilize questionnaires and sleep diaries to collect subjective reports of sleep^11^. In recent years, electronic surveys have been employed with limited scope and reach. Digital health methods can be used to measure variability in sleep and subsequent effects on daytime functioning over prolonged periods of time^12^. They also allow researchers to reach and enroll significantly larger study cohorts at a lower cost than traditional research methods^13,14^. Few studies carried out to date have explored the utility of assessing sleep health in this manner^15,16^.

The SleepHealth Mobile App Study (SHMAS) was an observational study developed using Apple’s ResearchKit (RK) (www.researchkit.org) platform, which has also been used by other recent digital health studies^17–21^. The primary goal of the SHMAS was to explore the relationship between sleep habits and daytime functioning using a novel digital health approach. Secondary goals included investigation of participant characteristics, evaluation of data validity and examination of user retention patterns and data-sharing preferences.

**Fig. 1:**
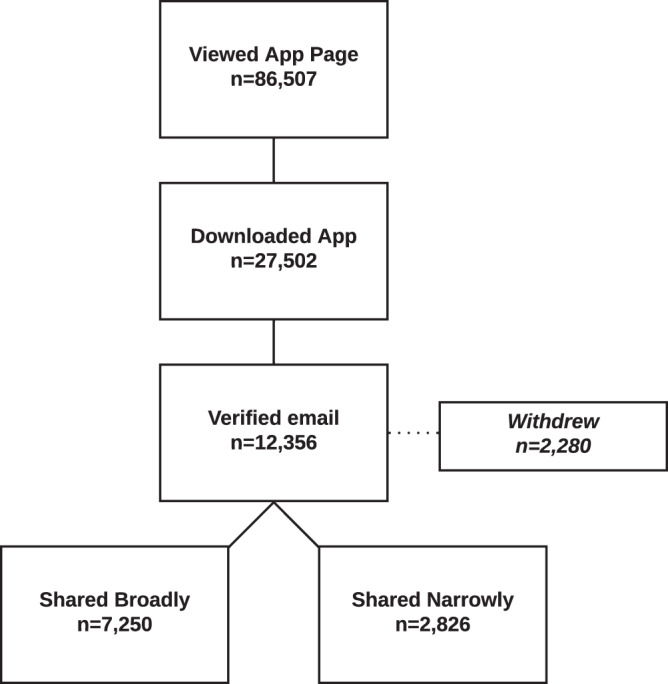
SHMAS Cohort Description. This figure shows the number of times the application was viewed and downloaded and the number of participants who made it all the way through to email verification, which indicated the start of the study. The figure then provides the number of participants who subsequently withdrew; of those who did not withdraw, the figure provides the number of participants who elected to share their data with qualified investigators and with only the study team.

The SHMAS was launched on the Apple App Store on March 2, 2016. The app store landing page was viewed 86,507 times and the application was downloaded 27,502 times. 12,356 participants provided consent to participate in the study through June 26, 2019 (Fig. 1). Study participants who agreed to share data broadly were enrolled in the study for an average of 935.4 days (SD = 355.1, range = 1–1212).

Upon enrollment in the study, baseline demographic and survey data were collected. Participants were instructed to actively monitor their sleep and activity patterns for a minimum of five days in a row every quarter. Participants were encouraged to share Apple Health compatible application and wearable data. Participants were also asked to select whether to share their data with the SleepHealth team only (narrowly) or with the team and other qualified researchers (broadly). In order to complete the consent process, participants had to make an active choice selecting the scope of data sharing as no default choice was specified^22^. The data described here and shared with the research community come from participants who chose to share their data broadly (7,250/12,356; 59%). The cohort consisted of primarily white (78%) males (79%) with a mean age of 37 years (SD = 13, range = 18–87). 40% of the sample completed a 4-year college degree or higher. Baseline demographic characteristics did not appear to meaningfully differ for participants when grouped by sharing status (Table 1), although the comparison yielded statistically significant differences. These statistically significant differences are likely attributable to a large sample size. Large-scale research studies like the SHMAS are testing novel ways to gather real-world data that may contribute to our understanding of long-term disease trajectories and disease management. Early studies have demonstrated the utility of digital health methods for collecting real-world data as well as their potential to fundamentally transform biomedical research^23,24^. However, concerns about engaging participants to provide long-term real-world data as well as the validity of inferences drawn from said data remain an issue^25^. To help address some of these challenges, there is a critical need to share the methods employed and lessons learned from studies carried out in this manner with the greater scientific community while following responsible data sharing guidelines.

**Table 1:** Baseline Demographics.

	Share Broadly *(Qualified researchers worldwide)*	Share Narrowly *(Study team and partners)*
*N*	7,250	2,826
**Age** (Mean (SD))*	36.6 (12.9)	38.5 (13.1)
**Gender** = Male (%)*	5,740 (79.2)	1,869 (66.1)
**Race** (%)*		
White	2,541 (77.9)	609 (67.4)
Asian	171 (5.2)	69 (7.6)
Black or African-American	93 (2.9)	61 (6.7)
Multiple Races	255 (7.8)	66 (7.3)
American Indian or Alaska Native	12 (0.4)	6 (0.7)
Prefer Not to Answer	36 (1.1)	37 (4.1)
Other	122 (3.7)	36 (4.0)
Native Hawaiian or Other Pacific Islander	7 (0.2)	4 (0.4)
**Ethnicity** (%)*		
Hispanic or Latino	370 (11.3)	113 (12.5)
Non-Hispanic or Latino	2,846 (87.2)	765 (84.6)
Unsure	25 (0.8)	7 (0.8)
Prefer Not to Answer	14 (0.4)	16 (1.8)
**Education** (%)*		
8th Grade or Less	9 (0.3)	3 (0.3)
Some High School	30 (0.9)	6 (0.7)
High School Graduate or GED	300 (9.2)	96 (10.6)
Some College	1057 (32.4)	260 (28.8)
College Graduate	852 (26.1)	241 (26.7)
More Than 4-year College Degree	975 (29.9)	270 (29.9)
Prefer Not to Answer	22 (0.7)	24 (2.7)
**Income** (%)*		
<10,000	175 (5.4)	45 (5.0)
10,000–49,999	767 (23.5)	182 (20.1)
50,000–99,999	782 (24.0)	200 (22.1)
100,000–149,999	525 (16.1)	127 (14.0)
150,000–199,999	254 (7.8)	51 (5.6)
200,000–249,999	140 (4.3)	37 (4.1)
250,000 and Above	218 (6.7)	48 (5.3)
Prefer Not to Answer	254 (7.8)	164 (18.1)
Unknown	121 (3.7)	36 (4.0)
**Marital Status** (%)*		
Now Married	1582 (48.5)	505 (55.9)
Never Married	977 (30.0)	214 (23.7)
Unmarried, But Living With a Partner	414 (12.7)	91 (10.1)
Divorced	192 (5.9)	59 (6.5)
Separated	46 (1.4)	8 (0.9)
Prefer Not to Answer	24 (0.7)	20 (2.2)
Widowed	20 (0.6)	5 (0.6)

Aggregate summary of baseline demographic characteristics (total number and percentage) for SHMAS study participants stratified by sharing status. Independent samples t-test and chi square tests yielded statistically significant between group differences for all characteristics, however the differences did not appear to be meaningful.

* Indicates p value < 0.001.

We hope that by making SHMAS data available in accordance with FAIR principles^26^, we will provide a rich dataset to researchers interested in studying sleep health. These data capture various sleep health constructs that can help to improve our understanding of sleep habits, sleep patterns, and their relationship with daytime alertness and health. We also hope to raise awareness and interest in sleep research in general and pave the way for more refined sleep studies in the future.

## Methods

### Participant onboarding

#### Overview

**Fig. 2:**
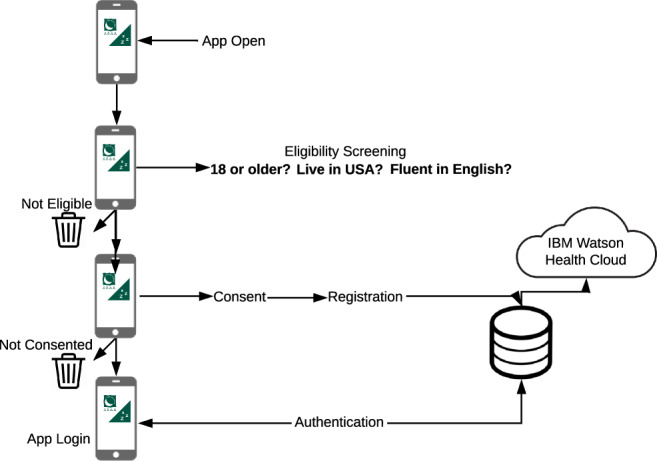
SHMAS Onboarding Process. This figure provides a graphical representation of the SHMAS onboarding process from first application use through enrollment and first login. It shows that there were several phases: screening, informed consent, registration and authentication.

The SHMAS was conducted remotely through an iPhone application. After installing the application, prospective participants were given a brief overview of the study and information about the study team (Fig. 2).

#### Eligibility

Prospective participants completed an in-application, three-item screener to determine their eligibility. The study was open to anyone with an iPhone who could install the application, was 18 years of age or older, lived in the United States, and could read and write fluently in English. There were no other criteria for inclusion in the study.

#### Participant expectations

**Fig. 3:**
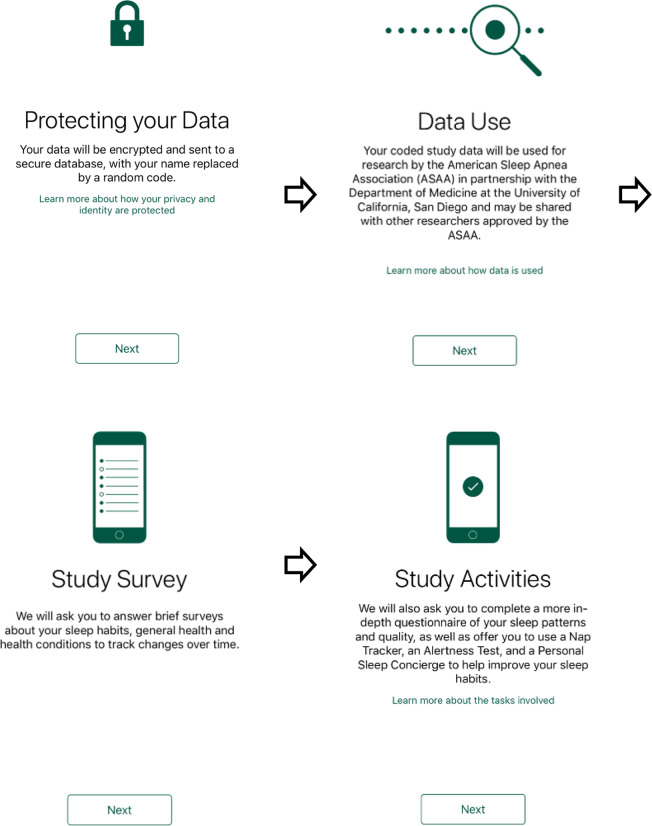
Screenshots of Onboarding Process. Prospective participants were given an overview of how their data would be protected and used. They were briefed on the types of sensor-based and passive Apple Health data that would be collected with their permission and also given an explanation of the types of surveys and daily activities that they would be expected to complete during the course of the study.

After successful completion of the eligibility screener, detailed information about the study was provided (Fig. 3). This included an explanation of how contributed data would be protected and used, the specific types of sensor-based and passive data that would be collected with permission, and the types of surveys and daily activities that prospective participants would be asked to complete.

#### Data sharing

Prospective participants were asked whether they wanted to share their study data for secondary research narrowly (With the SleepHealth team only) or broadly (with the SleepHealth team and other qualified researchers worldwide). No default sharing choice was selected. The study protocol and consent procedure were approved by the Western Institutional Review Board, Puyallup, WA (WIRB #20151042).

#### Understanding of study conditions

**Fig. 4:**
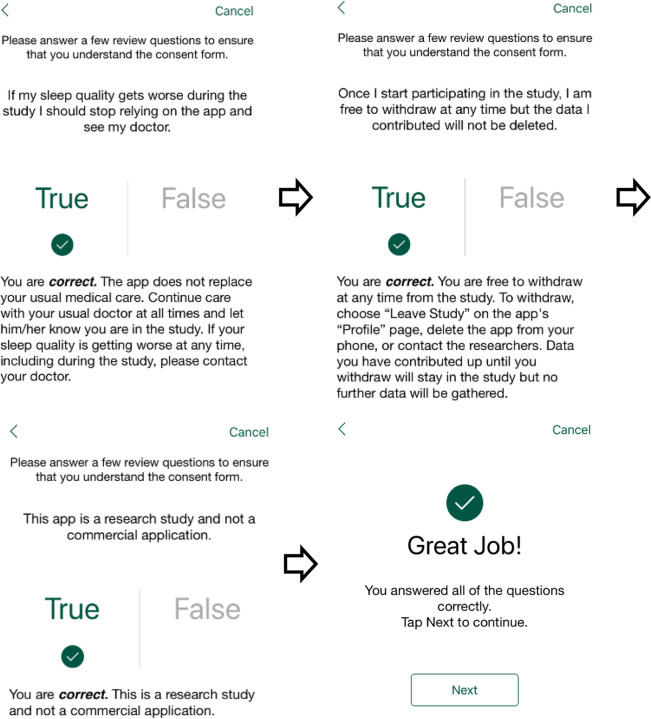
Screenshots of Informed Consent Test Questions. Prospective participants were asked three questions to ensure that they understood what their participation in research entailed: 1) If their sleep quality got worse during the study, they should stop relying on the application and see their doctor, 2) Once they started participating in the study, they were free to withdraw at any time but the data they contributed would not be deleted and 3) That the application was a research study and not a commercial application.

Potential participants were given contact information for the study team if they had any questions or concerns. They were then asked three questions about the information contained in the electronic informed consent process to ensure that they understood the terms of the study (Fig. 4). The three questions reinforced the following concepts: (1) if sleep quality got worse over the course of the study, they should contact their medical provider and not rely on the application; (2) that they could withdraw from the study at any time but the data that they contributed would not be deleted; and (3) that the application was a research study and not a commercial application. All three questions had to be answered correctly in order to proceed.

Prospective participants were shown an electronic copy of the consent form and asked to carefully review it. They were then asked to either click “Agree” or “Cancel.” If they clicked “Cancel,” they were not allowed to proceed any further and were not included in the study. If they clicked “Agree,” they were asked to confirm their decision by providing their first name, last name, and electronic signature.

#### Passive data

Participants were asked if they wanted to share passive data aggregated by Apple’s HealthKit framework^27^ with the study team. The default option was no sharing. Participants had the option to share as much or as little passive data as they wanted by either opting to share all categories of Healthkit data by selecting “Turn All Categories On” or by selecting individual data categories one by one.

#### Registration

To complete study registration, participants were asked to provide an email address, password, birthdate, and gender. They also selected a 4-digit passcode and chose permissions for receiving notifications. Participants had the ability to modify their notification permissions within the application at any time.

#### Account verification, consent form, withdrawal

Participants were required to verify their account via email and received a copy of their electronically signed consent form in PDF format. They could withdraw their consent at any time by selecting the “Leave Study” button from the study dashboard. Any data that participants provided up until the point of withdrawal were retained by the study team.

### Active data collection

The study consisted of two kinds of active data collection: one-time quarterly surveys and daily activities which were intended to be completed for a minimum of 5 out of 7 days each quarter. Participants had the option to complete the daily activities as many times as they wanted.

#### Surveys

The names of the surveys administered to participants in the study were: About Me (demographics); My Family (family medical history); My Health (general health and medical conditions); Research Interest (prior research experience/willingness to participate in future research studies); Sleep Habits (sleep duration and quality), and Sleep Assessment (sleep disorder symptoms and daytime functioning). The SHMAS utilized a combination of validated and adapted assessments that are commonly used in sleep research. Specific survey and questionnaire items are described in detail on the SleepHealth Public Researcher Portal.

#### Daily activities

**Fig. 5:**
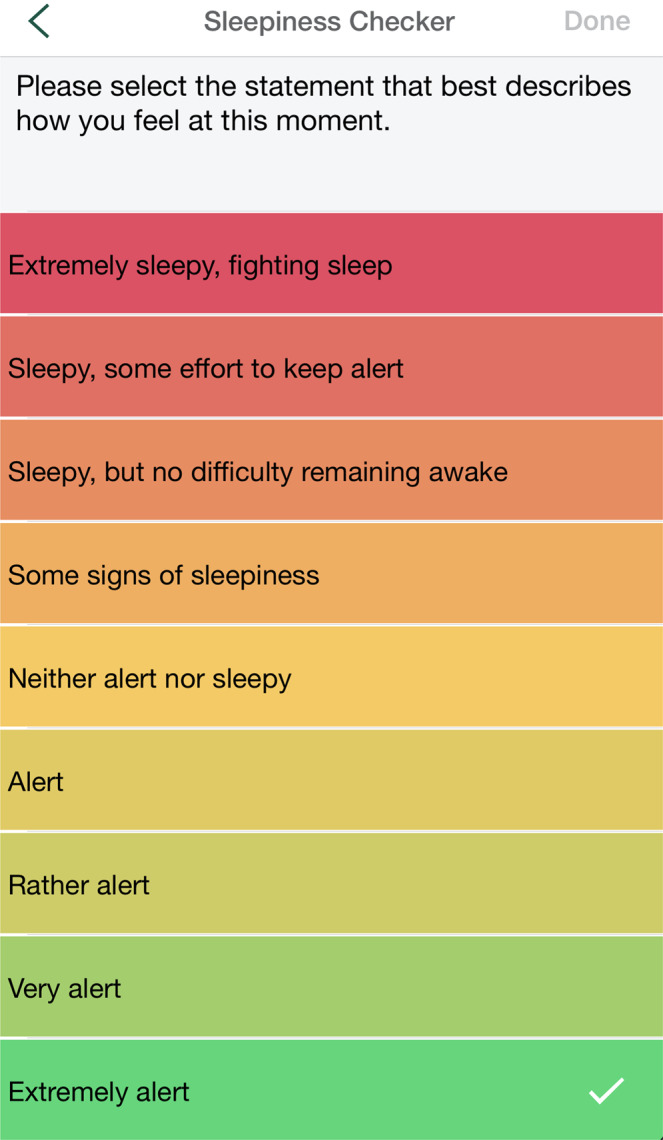
The Sleepiness Checker. The Sleepiness Checker could be used by participants to report their levels of sleepiness throughout the study. Participants received push notifications (if enabled) to complete it twice randomly throughout the day, but there was no limit on the number of times it could be completed.

**Fig. 6:**
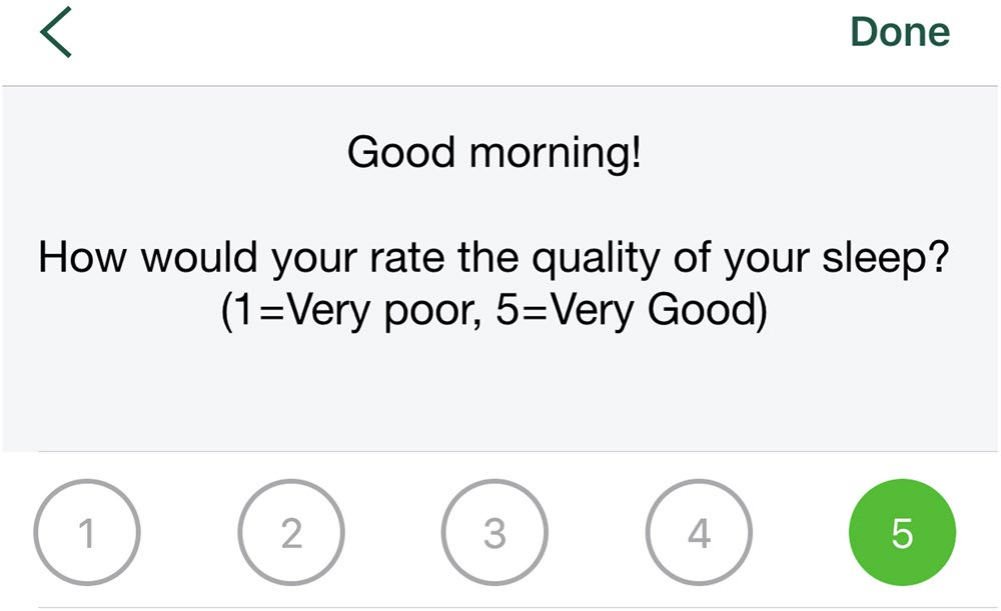
The Sleep Quality Checker. The Sleep Quality Checker could be used by participants to report their sleep quality for the previous night’s sleep. Participants received a push notification 1.5 hours after their reported wake-up times (if enabled) to provide this information, but it could be completed at any time on a given study day.

**Fig. 7:**
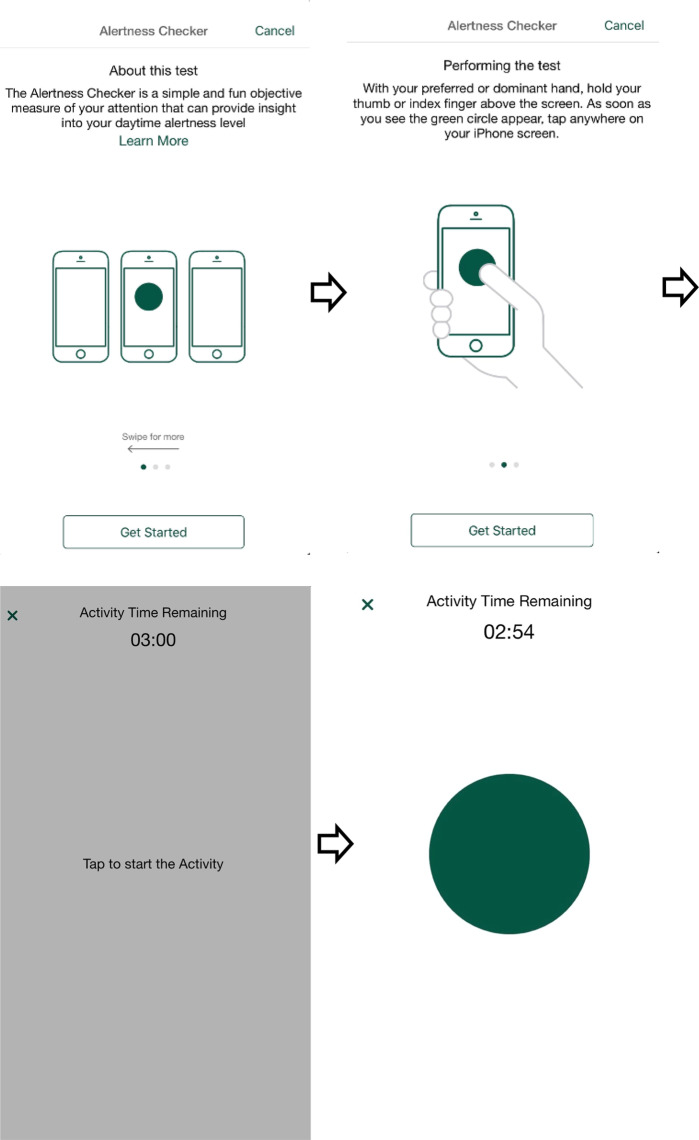
Alertness Checker Activity. The Alertness Checker was adapted from the 3-minute psychomotor vigilance task and was included as an objective measure of alertness. The stimulus was the green circle shown.

The AM and PM Check-in activities contained items from the Consensus Sleep Diary (CSD)^28^ and queried participants about their previous night’s sleep and their current day’s activities. The Sleepiness Checker activity was a direct adaptation of the Karolinska Sleepiness Scale (KSS)^29^. It utilized a 9-point Likert scale to evaluate subjective sleepiness and was anchored by “*extremely alert*” to “*extremely sleepy, fighting sleep*.” (Fig. 5). The KSS is a valid measure of daytime alertness/sleepiness and is highly correlated with EEG and other relevant behavioral metrics. The Sleep Quality Checker used a single-item from the CSD which utilized a 5-point likert scale to rate the previous night’s level of sleep quality. It was anchored by “*very poor*” to “*very good*.” (Fig. 6). The Alertness Checker was an objective, sensor-based task that was included in the SHMAS to assess sustained attention, which has been shown to be impaired with sleep loss^30^. It was a smartphone adaptation of the 3-minute Psychomotor Vigilance Task (PVT-B)^29,31^. The PVT-B has been demonstrated to be sensitive to reduced alertness resulting from sleep loss as well as improvements in alertness following recovery sleep^32,33^. The PVT-B has also been utilized in situations where it is impractical to use the original 10-minute PVT^34^. Participants were instructed to use the thumb or index finger of their dominant hand to tap their screen as quickly and accurately as possible in response to a target stimulus (Fig. 7). The Nap Tracker was a simple tool designed by the SHMAS study team that allowed participants to measure the timing and duration of naps. Participants could tap the screen to start the nap tracker at the beginning of their nap and tap again to stop the nap tracker upon awakening. They were also able to rate the quality of their naps on a scale ranging from “poor” to “excellent.”

#### Active task administration frequency

The SHMAS was designed to be a longitudinal observational study. The baseline onboarding surveys were gradually made available over the first three days of the study rather than all at once. This was done with the intention of making the baseline assessment less overwhelming. Table 2 outlines the survey and activity release schedule. Surveys were intended to be completed once during a baseline period and again at subsequent quarterly follow-ups. Daily activities were intended to supplement baseline and quarterly surveys, and users received notifications to complete them for 7-days in a row during the baseline period. After the baseline period ended, notifications automatically turned off by default. Users could re-enable notifications if desired, and were encouraged to continue to regularly use the daily activities to track their daytime sleepiness, sleep quality and objective vigilance.

**Table 2:** Schedule of Study Surveys and Activities.

Time	Task Name	Frequency	Duration	Timing of Notification
**Day 1**	AM Check-in	Daily	Ongoing	1 Hour After Wake-up Time
	Sleep Quality Checker	Daily	Ongoing	1.5 Hour After Wake-up Time
	Sleepiness Checker	Daily	Ongoing	Twice Randomly During Day
	Alertness Checker	Daily	Ongoing	Once Randomly During Day
	PM Check-in	Daily	Ongoing	1.5 Hours Before Sleep Time
	About Me	Once	Until Answered	N/A
	Sleep Habits	Once	Until Answered	N/A
	Nap Tracker	Daily	Ongoing	N/A
**Day 2**	My Family	Once	Until Answered	N/A
	Sleep Assessment	Once	Until Answered	N/A
**Day 3**	Research Interest	Once	Until Answered	N/A
	My Health	Once	Until Answered	N/A
**Day 7**	Completion of 7-day tasks	Once	Once	N/A

This table also provides the timing of the notifications for the daily activities. Notifications are reminders sent from the application to the phone to alert the participant to complete the activity.

### Passive healthkit data collection

Participants had the option to share HealthKit data with the study team during the onboarding process. For participants who had granted HealthKit sharing permissions, HealthKit data were passively collected in the background while the study application was open.

## Limitations

The SHMAS was only available to iPhone users residing in the United States. Location-based data were not collected for SHMAS participants in an effort to maintain participant privacy. Recruitment of participants for this iOS application based study may have introduced socioeconomic, gender and racial biases, which has been discussed in previous RK-based study publications^35,36^. Future digital health studies will benefit from being made available internationally and across iOS and Android platforms which will allow more diverse study cohorts to be recruited.

## Data Records

All coded data sets with associated metadata and documentation are stored and accessible via the Synapse platform by qualified researchers (https://www.synapse.org/sleephealth). Detailed instructions describing the process of requesting access to data can be found on the SleepHealth Public Researcher Portal under the “Accessing SleepHealth Data” page.

Please see Tables 3 and 4 for a breakdown of surveys and daily activities completed by study participants who agreed to share their data broadly^37–49^. Table 5 describes the kinds of HealthKit data that were provided by study participants over the course of the study^50–58^.

**Table 3:** Available Survey Data.

Survey Name	Availability	Description	DOI	Unique Participants	Total Surveys Completed
Onboarding Demographics	Available during consent	Basic demographics (weight, sex, height, age)	10.7303/syn22264195.1^37^	7,250	8,130
About Me	Available on Day 1	More detailed demographic information	10.7303/syn22264269.1^38^	3,262	3,448
Sleep Habits	Available on Day 1	Sleep duration and quality	10.7303/syn22264326.1^39^	3,155	3,303
My Family	Available on Day 2	Family medical history	10.7303/syn21966449.1^40^	2,760	3,003
Sleep Assessment	Available on Day 2	Sleep disorder symptoms/daytime function	10.7303/syn21966546.1^41^	2,228	2,325
Research Interest	Available on Day 3	Prior research experience/willingness to participate in research	10.7303/syn21966541.1^42^	2,192	2,359
My Health	Available on Day 3	Health and medical conditions	10.7303/syn22264395.1^43^	1,478	1,551

This table provides the number of unique participants and number of total survey administrations completed.

**Table 4:** Available Daily Activity Data.

Activity Name	Availability	Description	DOI	Unique Participants	Total Activities Completed
AM Check-in	Available on Day 1	Question about prior night’s sleep	10.7303/syn22264398.1^44^	5,266	49,480
PM Check-in	Available on Day 1	Questions about current day	10.7303/syn22264410.1^45^	4,313	27,380
Sleep Quality Checker	Available on Day 1	Single item from Consensus Sleep Diary (CSD)	10.7303/syn22264412.1^46^	4,566	42,567
Sleepiness Checker	Available on Day 1	Karolinska Sleepiness Scale (KSS)	10.7303/syn22264418.1^47^	4,636	49,188
Nap Tracker	Available on Day 1	Simple tool to assess timing, duration and quality of naps	10.7303/syn22264428.1^48^	592	2,093
Alertness Checker	Available on Day 1	3-minute Psychomotor Vigilance Task (PVT-B)	10.7303/syn22271040.1^49^	4,031	18,915

This table provides the number of unique participants and number of total daily activity administrations completed.

**Table 5:** Available HealthKit Data.

Data Type	Description	DOI	Unique Participants	Distinct Days of Data
HealthKit.StepCount	Step count	10.7303/syn21963204.1^50^	2,592	16,825
HealthKit.Height	Height	10.7303/syn21963203.1^51^	1,933	2,037
HealthKit.HeartRate	Heart rate	10.7303/syn21963196.1^52^	2,012	14,646
HealthKit.FlightsClimbed	Flights of stairs climbed	10.7303/syn21963195.1^53^	1,537	3,896
HealthKit.DistanceWalkingRunning	Distance walked or ran	10.7303/syn21962791.1^54^	2,480	17,108
HealthKit.BodyMass	Body mass	10.7303/syn21962784.1^55^	2,014	2,575
HealthKit.BasalEnergyBurned	Basal energy burned	10.7303/syn21962783.1^56^	752	6,412
HealthKit.AppleExerciseTime	Time exercised	10.7303/syn21962782.1^57^	467	1,591
HealthKit.ActiveEnergyBurned	Active energy burned	10.7303/syn21962781.1^58^	563	9,828

This table provides the number of unique participants and number of distinct days that participants contributed each type of corresponding HealthKit data. Participants had the option to share their HealthKit data after enrolling in the study.

## Technical Validation

The data provided on the SleepHealth Public Researcher Portal (www.synapse.org/sleephealth) come from participants who agreed to share broadly with the study team and qualified researchers worldwide. All data are self-reported unless otherwise indicated, and should be treated as such. The study portal also contains passively collected Apple HealthKit data from participants who consented to data sharing.

Potentially sensitive data such as birthdates and free-text responses were excluded in order to safeguard participant privacy. In order to protect participants with potentially identifiable traits, self-reported height and weight data that fell outside thresholds established in a previously published data descriptor manuscript (height <60 or >78 inches, weight <80 or >350lbs)^35^ were excluded and set to ‘CENSORED’ in the corresponding data frames. Despite the fact that all study participants affirmed that they were 18 years of age or older at the time of study enrollment, some participants later provided dates of birth which indicated that they were under 18 years old. Data contributed by such participants were excluded. Additionally, data from test accounts that were created during the initial study period were also excluded in order to maximize data quality.

The raw passive data collected through Apple’s HealthKit framework were cleaned extensively. Specifically, data that contained sensitive and potentially identifiable information such as participant location, and device name (which in some cases contained participant names) were excluded. Finally, the subset of HealthKit data types that were shared by at least 1% of the participants in the HealthKit data sharing cohort were selected for broad release.

## Usage Notes

Governance structures have been put in place in order to respect the balance between the desire of participants to share their data with qualified researchers and the potential risk of re-identification of participant data. To obtain access to these data, researchers must complete the following steps:

1. Become a Synapse Certified User with a validated user profile.
2. Submit a 1–3 paragraph Intended Data Use statement to be posted publicly on Synapse.
3. Agree to comply with the data-specific Conditions for Use when prompted:

- You confirm that you will not attempt to re-identify research participants for any reason, including for re-identification theory research.
- You reaffirm your commitment to the Synapse Awareness and Ethics Pledge.
- You agree to abide by the guiding principles for responsible research use and data handling as described in the Synapse Governance documents (http://docs.synapse.org/articles/governance.html).
- You commit to keeping these data confidential and secure.
- You agree to use these data exclusively as described in your submitted Intended Data Use statement.
- You understand that these data may not be used for commercial advertisement or to re-contact research participants.
- You agree to report any misuse or data release, intentional or inadvertent to the ACT within 5 business days by emailing act@sagebase.org.
- You agree to publish findings in open access publications.
- You promise to acknowledge the research participants as data contributors and the study investigators on all publication or presentation resulting from using these data as follows:

“These data were contributed by the participants of the SleepHealth Mobile App Study (SHMAS), which was sponsored by the American Sleep Apnea Association with scientific oversight by Carl Stepnowsky, PhD, as described in Synapse: https://www.synapse.org/sleephealth.”

## Data Availability

The SHMAS was built using Apple’s open source ResearchKit framework (https://github.com/researchkit/researchkit) and AppCore (https://github.com/ResearchKit/AppCore). The SHMAS source code is available upon request. Participant data hosting was handled by IBM Watson Health Cloud (https://www.ibm.com/watson-health/), a data collection solution where participant data were stored before being imported into Sage Synapse. The code used to process and clean the raw study data can be found on GitHub (https://github.com/apratap/SleepHealth_Data_Release). Suggested thresholds for excluding outliers are included in the code but commented out.
